# The economic burden of systemic sclerosis related pulmonary arterial hypertension in Australia

**DOI:** 10.1186/s12890-019-0989-1

**Published:** 2019-11-27

**Authors:** Kathleen Morrisroe, Wendy Stevens, Joanne Sahhar, Gene-Siew Ngian, Nava Ferdowsi, Dylan Hansen, Shreeya Patel, Catherine L. Hill, Janet Roddy, Jennifer Walker, Susanna Proudman, Mandana Nikpour

**Affiliations:** 1 Department of Medicine, The University of Melbourne at St Vincent’s Hospital (Melbourne), 41 Victoria Parade, Fitzroy, Victoria 3065 Australia; 2 Department of Rheumatology, St Vincent’s Hospital (Melbourne), 41 Victoria Parade, Fitzroy, Victoria 3065 Australia; 3 Department of Medicine, Monash University, Clayton and Monash Health, 246 Clayton Road, Clayton, Victoria 3168 Australia; 4 Rheumatology Unit, Royal Adelaide Hospital, North Terrace, Adelaide, SA 5000 Australia; 5 Rheumatology Unit, The Queen Elizabeth Hospital, Woodville Road, Woodville, SA 5011 Australia; 6 Discipline of Medicine, University of Adelaide, Adelaide, SA 5000 Australia; 7 Department of Rheumatology, Royal Perth Hospital, Perth, Australia; 8 Rheumatology Unit, Flinders Medical Centre (Adelaide), Flinders Drive, Bedford Park, South Australia 5042 Australia

**Keywords:** Systemic sclerosis, Scleroderma, Pulmonary arterial hypertension, Healthcare utilization and associated direct cost, Economic Burden

## Abstract

**Background:**

To quantify the financial cost of pulmonary arterial hypertension (PAH) in systemic sclerosis (SSc).

**Methods:**

Healthcare use was captured through data linkage, wherein clinical data for SSc patients enrolled in the Australian Scleroderma Cohort Study were linked with hospital, emergency department (ED) and ambulatory care databases (MBS) for the period 2008–2015. PAH was diagnosed on right heart catheter according to international criteria. Determinants of healthcare cost were estimated using logistic regression.

**Results:**

Total median (25th–75th) healthcare cost per patient (including hospital, ED and MBS cost but excluding medication cost) for our cohort during 2008–2015 was AUD$37,685 (18,144-78,811) with an annual per patient healthcare cost of AUD$7506 (5273-10,654). Total healthcare cost was higher for SSc-PAH patients compared with those without PAH with a total cost per patient of AUD$70,034 (37,222-110,814) vs AUD$34,325 (16,093 – 69,957), *p* < 0.001 respectively with an annual excess healthcare cost per PAH patient of AUD$2463 (1973-1885), *p* < 0.001. The cost of SSc-PAH occurs early post PAH diagnosis with 89.4% utilizing a healthcare service within the first 12 months post PAH diagnosis with an associated cost per patient of AUD$4125 (0–15,666). PAH severity was the main significant determinant of increased healthcare cost (OR 2.5, *p* = 0.03) in our PAH cohort.

**Conclusions:**

Despite SSc-PAH being a low prevalence disease, it is associated with significant healthcare resource utilization and associated economic burden, predominantly driven by the severity of PAH.

## Background

Systemic sclerosis (SSc) is a chronic multisystem autoimmune disease characterized by vasculopathy and fibrosis [1] occurring with a worldwide prevalence of 7–489/million and an incidence of 0.6–122/million/year [1]. As there is no cure for SSc and few effective disease modifying agents, SSc is associated with significant morbidity, mortality and reduced health related quality of life (HRQoL) [2]. Furthermore, SSc is associated with a substantial healthcare cost with an annual health service utilisation cost per patient, including hospitalisations, emergency care, ambulatory visits and medication use, of AUD$11,607.07 (direct cost) [3], predominantly driven by the presence of cardiopulmonary involvement including pulmonary arterial hypertension (SSc-PAH) and interstitial lung disease (SSc-ILD) [3–5].

SSc-PAH, occurring with a prevalence of 10–13% in SSc [6, 7], is the leading cause of SSc-related mortality [8], with a standardized mortality ratio (SMR) of 5.8 (95%CI 4.3–7.8) and 15.2 years of life lost (YLL) [9]. PAH is characterized by abnormal proliferation, vasoconstriction and in-situ thrombosis of the pulmonary vasculature, leading to elevated pulmonary vascular resistance (PVR), resulting ultimately in right heart failure and death [10]. Despite annual screening for PAH and the availability of specific PAH vasodilator therapies, the median (25th–75th) survival time from PAH diagnosis is only 4.0 (2.2–6.2) years [9]. Predictors of mortality include worse World Health Organization Functional Class (WHO-FC), the presence of a pericardial effusion, advancing age (> 60 years), male gender and a worse hemodynamic profile at PAH diagnosis (defined by increased mean pulmonary arterial pressure (PAP), right atrial pressure (RAP) and PVR, and a reduced cardiac index (CI)), while the use of combination PAH therapy confers a survival advantage over monotherapy [9, 11]. In Australia, the government only subsidizes monotherapy if prescribed by a physician in a government designated PAH treatment centre with combination therapy occurring through hospital or pharmaceutical company compassionate access, or at patients’ own expense.

Given the prevalence of SSc-PAH and its poor prognosis, it is surprising that studies on healthcare utilization and associated economic burden specific to SSc-PAH are scarce with only one retrospective cohort study assessing all-cause healthcare cost in SSc patients with lung involvement including only 108 SSc-PAH patients [4]. Studies on healthcare utilization and associated cost in general PAH, not specific to Group 1 PAH that encompasses SSc-PAH, are more prevalent and have highlighted the importance of a point-in-time measurement of the WHO-FC as a predictor of PAH progression, survival, healthcare utilization and associated cost [12, 13]. One might therefore deduce that by improving overall survival and reducing or maintaining WHO-FC through the initiation of PAH specific therapy one might alter the associated healthcare burden (predominantly through reduced hospitalizations and ambulatory care visits) and cost. However, this has only been reported in a couple of studies preformed in the US in patients with general PAH [14–16].

Therefore, we sought to quantify healthcare utilization in SSc-PAH and its associated direct cost by means of data linkage and to determine the presence, if any, of modifiable factors. Efficient usage of healthcare budgets is critical for the maintenance of a sustainable healthcare system and relies on economic studies, such as ours, to provide real life cost figures to clinicians and health policy makers to enable the appropriate allocation of resources and to evaluate health policies and interventions.

## Methods

Consecutive SSc patients from four Australian states [Victoria (VIC), South Australia (SA), Western Australia (WA) and Tasmania (TAS)] prospectively enrolled in the Australian Scleroderma Cohort Study (ASCS), a multi-center study of clinically important outcomes in SSc, were included. The ASCS database collects comprehensive demographic and disease-related data on an annual basis. Written consent was obtained from all patients at recruitment and ethical approval was obtained from all participating hospitals (St. Vincent’s Hospital, Melbourne, Monash Health, Royal Adelaide Hospital, Royal Perth Hospital and Royal Hobart Hospital, Tasmania).

### Inclusion and exclusion criteria

We included all adult (≥18 years) SSc patients recruited in the ASCS between January 2008 (cohort inception) and December 2015 (when data linkage occurred). All patients fulfilled the American College of Rheumatology / European League Against Rheumatism Classification criteria for SSc [17]. To ensure that we only evaluated the cost associated with WHO Group 1 PAH in our SSc cohort, we excluded those with Group 1 PAH and co-existing ILD with a FVC < 70% and an abnormal high-resolution computer tomography (HRCT) of the chest. Ventilation perfusion scanning was preformed to exclude pulmonary hypertension due to chronic thromboembolism.

### ASCS clinical data

All ASCS patient undergo annual screening for PAH and ILD with a transthoracic echocardiogram (TTE) and respiratory function test (RFT). PAH was diagnosed by right heart catheterization (RHC) according to international criteria [18]. Severe PAH was defined based on presence of four of the following PAH severity criteria: WHO-FC IV, pericardial effusion, six-minute walk distance (6MWD) < 300 m, RAP on RHC of > 15 mmHg and CI of < 2 L/min/m^2^ [19]. Mild ILD was defined by characteristic fibrotic changes on high-resolution computed tomography (HRCT) lung (< 10% abnormal lung involvement) and a forced vital capacity of > 70% on respiratory function tests [17, 20]. Monotherapy is treatment with a single PAH specific therapy (endothelin receptor antagonists (ERA), phosphodiesterase-5-inhibitors (PDE5) or prostacyclin analogues) and was prescribed at the discretion of the managing physician(s), while combination therapy is treatment with more than one specific PAH agent from different classes at one time. Patient status (alive or dead) was censored in January 2016.

### Data linkage

Healthcare utilization was captured by means of a comprehensive data linkage that we have previously described [21].

### Healthcare utilization and costing methodology

The method of calculating healthcare utilization and costing has been previously described [21]. The most common reasons for hospital admission were noted for admissions lasting more than one day in order avoid including recurring day admissions for example for intravenous infusions and haemodialysis. The cost of PAH specific vasodilator therapies was calculated from the PBS Dispensed Price for Maximum Quantity (DPMQ) paid for the standard dose of each medication, which is the cost the government contributes towards each medication dispensed. However, this cost was not included in the total healthcare cost calculation so as to allow the equitable comparison of healthcare service use in those with and without PAH. It is included as a Additional file Table.

### Statistical analysis

Data are presented as mean ± standard deviation (SD) for normally distributed, median (25th–75th) for non-normally distributed continuous variables, and as number (percentage) for categorical variables. Differences in frequency were tested using chi-square and Fisher’s exact tests. To determine the associations of different PAH specific variables with healthcare utilization and associated cost univariable and multivariable logistic regression were used. Variables with a *p*-value < 0.05 in univariable regression or variables deemed to be of clinical significance to the outcome with a p-value < 0.20 were included in the multivariable logistic regression analysis. A subgroup analysis to determine healthcare costs between those with and without PAH was preformed using propensity score matching. A two-tailed *p* value of 0.05 or less was considered statistically significant. All statistical analyses were performed using STATA 14.0 (StataCorp LP, College Station, TX, USA).

## Results

### Patient characteristics

Our study cohort consisted of 1128 SSc patients contributing 5527.2 person years of follow-up. Of these, 153 (13.6%) developed PAH over a mean follow-up of 4.6 ± 2.6 years with a mean age at diagnosis of 63.4 ± 10.4 years. PAH patients, compared with those without PAH, were older at SSc onset (49.3 vs 45.7 years, *p* = 0.004) with a longer disease duration (14.5 vs 10.6 years) and were more likely to be anti-centromere antibody (ACA) positive (50.3% vs 41.6%, *p* = 0.01), have digital ulcers (57.3% vs 47.2%, *p* = 0.02), telangiectasia (92.2% vs 83.6%, *p* = 0.01) and calcinosis (58.2% vs 39.9%, *p* < 0.001) (Table 1). Furthermore, they were more likely to be in WHO-FC III or IV (86% vs 28.0%, *p* < 0.001), have a low 6MWD (289.6 m vs 439.1 m, *p* < 0.001) and have a history of angina (26.2% vs 12.0%, *p* < 0.001) (Table 1).

**Table 1:** Characteristics of SSc patients by PAH status

Patient characteristics (*n*=1,128)	PAH	No PAH	*p* value
	mean ± SD or n(%)	mean ± SD or n(%)	
Number of patients	153 (13.6%)	975 (86.4%)	
Demographics			
Age on SSc onset^a^, years	49.3 ± 13.9	45.7 ± 13.9	0.004
Disease duration at recruitment, years	14.5 ± 11.9	10.6 ± 9.9	<0.01
Female	134 (87.6%)	794 (81.4%)	0.06
Limited disease subtype	115 (75.2%)	684 (73.4%)	0.65
Caucasian Ethnicity	142 (94.7%)	839 (94.8%)	0.95
Follow-up, years	4.6 ± 2.6	4.9 ± 2.5	0.16
Alive at censorship	68 (44.4%)	707 (72.5%)	<0.01
Autoantibody profile			
Anti-centromere pattern ANA	77 (50.3%)	406 (41.6%)	0.01
Scl 70 +ve	14 (9.2%)	132 (13.5%)	0.02
RNA polymerase III +ve	12 (7.9%)	87 (8.9%)	0.07
Clinical manifestations^b^			
Digital ulcers ever	87 (57.3%)	440 (47.2%)	0.02
Telangiectasia ever	141 (92.2%)	815 (83.6%)	0.006
Calcinosis ever	89 (58.2%)	389 (39.9%)	<0.01
GIT involvement	133 (86.9%)	802 (82.3%)	0.15
Renal Crisis	2 (1.3%)	31 (3.3%)	0.18
Pericardial Effusion	28 (41.3%)	124 (12.3%)	<0.01
Mild ILD (FVC>70%)	42 (27.5%)	208 (21.3%)	0.09
6MWD	289.6 ±115.3	439.1 ± 118.2	<0.01
WHO Functional Class			
Class I	2 (1.3%)	275 (30.6%)	<0.01
Class II	19 (12.6%)	372 (41.4%)	
Class III	83 (55.0%)	231 (25.7%)	
Class IV	47 (31.1%)	21 (2.3%)	
Brain Natriuretic Peptide	295 ± 140	84.9 ± 18.0	<0.01
N-terminal pro b-type Natriuretic Peptide	1799 ± 1542	375 ± 110	0.03
Co-morbidities			
Angina	32 (26.2%)	121 (12.0%)	<0.01
CVA	7 (11.1%)	146 (13.7%)	0.56
Diabetes Mellitus	14 (15.7%)	139 (13.4%)	0.53
COAD	23 (16.5%)	130 (13.1%)	0.27
Current smoker	15 (10.4%)	138 (14.1%)	0.19

Abbreviations: pulmonary arterial hypertension (PAH), interstitial lung disease (ILD), gastrointestinal tract (GIT), six minute walk distance (6MWD), world health organization (WHO), health assessment questionnaire (HAQ), scleroderma HAQ (sHAQ), hypertension (HTN), cerebrovascular accident (CVA), peripheral vascular disease (PVD), chronic obstructive airways disease (COAD)

^a^disease duration defined as from first non-Raynaud’s disease manifestation,

^b^clinical manifestations defined as present if ever present from SSc diagnosis

PAH patients were predominantly Caucasian (94.7%) females (87.6%), with limited disease subtype (75.2%) (Table 1). Of those with autoantibody data, 77 (50.3%) were positive for ACA, 14 (9.2%) were antitoposomerase-1 (Scl-70) positive and 12 (7.9%) were RNA Polymerase III (RNAP) antibody positive (Table 1). At the time of PAH diagnosis, the mean 6MWD was 329.3 ± 109.9 m, over three quarters of patients were in WHO-FC III or IV (62.5% in WHO-FC III, 9.8% in WHO-FC Class IV), 11.8% had a pericardial effusion and 66% fulfilled the criteria for severe PAH (Table 2). Survival in those with PAH was 4.8 ± 3.0 years from PAH diagnosis (4.3 ± 2.7 years for those who died and 5.3 ± 3.3 years in those still alive at censorship) (Table 2). One third of patients were treated with combination PAH therapy with the remainder treated with monotherapy (Table 2). PAH patients compared to those without PAH were more likely to be prescribed an anticoagulant and a diuretic (56.5% vs 19.9%, *p* < 0.001 and 29.9% vs 7.1%, *p* < 0.001).

**Table 2:** Characteristics at PAH diagnosis

Patient characteristics at PAH diagnosis	mean ± SD or n(%)	p-value
Demographics		
Age at PAH diagnosis, years	63.4 ± 10.4	
Disease duration at PAH diagnosis^a^, years	14.1 ± 11.6	
WHO Functional Class at diagnosis		
Class I	6 (4.5%)	
Class II	32 (2.4%)	
Class III	82 (62.5%)	
Class IV	13 (9.8%)	
Hemodynamic measurements at PAH diagnosis		
6MWD,m	329.3 ±109.9	
mRAP, mmHg	7.9 ± 3.8	
mPAP, mmHg	33.9 ± 10.9	
PAWP, mmHg	10.9 ± 4.0	
mCI, L/min/m2	2.9 ± 1.3	
PVR, Wood Units	4.7 ± 2.8	
Pericardial effusion at PAH diagnosis	18 (11.8%)	
Mean DLCO	48.9 ± 15.5	
Mean DLCO/VA, % predicted mL/min/mmHg	58.5 ± 19.6	
Severe PAH^b^	101 (66.0%)	
Overall survival	4.8 ± 3.0	0.04
Time to death from PAH diagnosis, years(n=77)	4.3 ± 2.7	
Survival from PAH diagnosis, years (n=68)	5.3 ± 3.3	

Abbreviations: pulmonary arterial hypertension (PAH), six minute walk distance (6MWD), mean pulmonary arterial pressure (mPAP), mean right atrial pressure (mRAP), pulmonary vascular resistance (PVR), cardiac index (CI), pulmonary arterial wedge pressure (PAWP), diffusing capacity of the lungs for carbon monoxide (DLCO), DLCO divided by the alveolar volume (DLCO/VA), world health organization (WHO)

^a^disease duration defined as from first non-Raynaud’s disease manifestation,

^b^severe PAH defined by the presence of WHO Functional Class IV, presence of a pericardial effusion, 6MWD <300m, right atrial pressure on right heart catheter of >15 and cardiac index of <2.

### Healthcare utilization and associated direct cost

#### Hospital admission and cost

Within our cohort, 81.8% of patients were admitted to hospital at least once during 2008–2015, more commonly for patients with SSc-PAH compared with those without PAH (92.2% vs 80.2%, *p* < 0.001). The annual per patient median admission frequency was 5 (2–11) and average length of stay (LOS) was 2.1 (1.1–4.1) days, higher for those with SSc-PAH that those without (admission frequency 5 (3–10) vs 4 (2–11), *p* < 0.001 and LOS 3.5 (1.8–4.9) vs 1.9 (1.1–1.9), *p* < 0.001 respectively). The primary admission diagnosis for SSc-PAH patients was PAH followed by heart failure, while for those without PAH, a complication of their systemic sclerosis followed by pneumonia were the most common reasons for admission (Table 3).

**Table 3:** Healthcare utilisation in SSc by PAH status between 2008-2015

Characteristics per patient	Overall n (%) or mean±SD Median (IQR)	PAH n (%) or mean±SD Median (IQR)	No PAH n (%) or mean±SD Median (IQR)	*p*-value
Hospitalisations				
Patient number	923	141	782	
% of patients admitted to hospital	923/1128 (81.8%)	141/153 (92.2%)	782/975 (80.2%)	<0.001
Median admissions per patient (2008-2015)	5 (2-11)	5 (3-10)	4 (2-11)	<0.001
Median annual hospital admissions per patient	3.2 ± 6.4	4.4 ± 6.2	2.9 ± 6.4	<0.001
Mean annual	1.7 (1-3.3)	3 (1.7-4.5)	1.6 (1-3)	
Average LOS per patient per admission	2.1 (1.1-4.1)	3.5 (1.8-4.9)	1.9 (1.1-1.9)	<0.001
	3.1 ± 3.1	3.9 ± 3.3	2.9 ± 3.1	
Reason for admission^a^				
1.	Systemic sclerosis(10.9%)	PAH (9.7%)	Systemic sclerosis (7.8%)	
2.	Pneumonia (5.4%)	Heart Failure (6.4%)	Pneumonia (5.2%)	
3.	PVD (4.5%)	Pneumonia (4.6%)	PVD (3.7%)	
4.	Heart Failure (2.5%)	Systemic sclerosis	Iron deficiency anaemia	
5.	Iron deficiency anaemia	(4.1%)	(2.4%)	
	(1.6%)	Acute LRTI (2.3%)	Chronic ulcer (1.4%)	
Emergency Department Presentations				
Patient number	734	122	612	
% of patients presenting to ED	734/1128 (65.1%)	122/153 (79.8%)	612/975 (62.8%)	<0.001
Median presentation per patient (2008-2015)	7 (3-12)	9 (5-14)	6 (2-12)	<0.001
Mean number	8.9 ± 7.7	10.0 ± 6.8	8.6 ± 7.9	<0.001
Median annual presentations per patient	2 (1-3)	2 (1-4)	2 (1-3)	<0.001
Mean annual presentations per patient	2.6 ± 2.0	3.1 ± 2.3	2.5 ± 1.9	<0.001
Top 5 reasons for ED presentation				
1.	Chest pain (4.5%)	Chest pain (4.9%)	Chest pain (4.3%)	
2	Dyspnoea (2.5%)	Dyspnoea (3.8%)	Abdominal pain (2.6%)	
3.	Abdominal pain (2.4%)	Acute LRTI (3.6%)	Dyspnoea (2.1%)	
4.	Acute LRTI (2.2%)	Lobar pneumonia (3.4%)	Acute LRTI (1.8%)	
5.	Lobar pneumonia (2.1%)	Intestinal obstruction (2.8%)	Lobar pneumonia (1.8%)	
Medical Benefit Schedule (MBS)				
Patient number	1,081	151	930	
% of patients utilizing an MBS service	1,081/1,128 (95.8%)	151/153 (98.7%)	930/975 (95.4%)	0.05
Median services utilized per patient (2008-2015)	557 (378-863)	761 (512-1,116)	523 (352-1116)	<0.001
Mean number	670.2 ± 451.6	873.9 ± 502.6	623.4 ± 425.5	<0.001
Median annual MBS services utilized per patient	76 (47-125)	108 (71-162)	69 (43-113)	<0.001
Mean annual MBS services utilized per patient	68.5 ± 56.9	100.9 ± 61.8	63.3 ± 54.4	<0.001
Top 5 MBS services utilized				
1.	Pathology (42.9%)	Pathology (47.8%)	Pathology (41.8%)	
2.	Professional visit (30.1%)	Professional visit	Professional visit (30.8%)	
3.	Allied Health (14.2%)	(26.9%)	Allied Health (14.2%)	
4.	Diagnostic imaging (5.4%)	Allied Health (14.1%)	Diagnostic imaging (5.5%)	
5.	Therapeutic procedures (4.4%)	Diagnostic imaging (4.9%)	Therapeutic procedures (4.5%)	
		Therapeutic procedures (3.9%)		

Abbreviations: medical benefits schedule (MBS), length of stay (LOS), emergency department (ED), lower respiratory tract infection (LRTI), pulmonary arterial hypertension (PAH), peripheral vascular disease (PVD), patient (pt)

^a^these hospital admissions are the most frequent admission diagnosis for admissions lasting more than one day in order avoid including recurring day admissions for intravenous infusions and haemodialysis

The total hospital admission cost per patient for our cohort during 2008–2015 was AUD$81,530 (42,637-175,641) with a median (25th–75th) annual admission cost per patient of AUD$3850 (2387-5981) (Table 4). These costs were higher for SSc-PAH patients compared with those without PAH with a total admission cost per patient of AUD$94,991 (56,866-178,464) vs AUD$79,052 (37,698 – 175,264), *p* < 0.001 and mean annual admission cost per patient of AUD$4802 (3344-6948) vs AUD$3654 (2251-5737), *p* < 0.001 (Table 4).

**Table 4:** The economic burden associated with healthcare utilization in SSc by PAH status

Characteristics per patient	Overall mean±SD, median (IQR) AUD$	PAH mean±SD, median (IQR) AUD$	No PAH mean±SD, median (IQR) AUD$	*p*-value
Hospitalisations				
Total admission cost per patient (2008-2015)	81,530 (42,637-175,641)	94,991 (56,866-178,464)	79,052 (37,698 – 175,264)	<0.01
Mean annual admission cost per patient	4,730 ± 4,348	5,405 ± 3,144	4,609 ± 4,522	<0.01
Median annual admission cost per patient	3,850 (2,387-5,981)	4,802 (3,344-6,948)	3,654 (2,251-5,737)	<0.01
Emergency Department				
Total ED cost per patient (2008-2015)	3,047 (1,266-5,465)	3,676 (2,099-5,699)	2,836 (1,034-5,279)	<0.01
	3823 ± 3349	4,234 ± 2,880	3,718 ± 3,451	<0.01
Mean annual ED cost per patient	531 ± 663	874 ± 900	477 ± 600	<0.01
Median annual ED cost per patient	422 (0-748)	655 (421-1,009)	411 (0-718)	<0.01
Medical Benefit Schedule (MBS)				
Total MBS cost per patient (2008-2015)	23,568 (15,987 – 35,111)	27,531 (19,493-39,738)	22,861 (15,359-33,842)	<0.01
Mean annual MBS cost per patient	3,038 ± 2,720	4,056 ± 3,175	2,878 ± 2,607	<0.01
Median annual MBS cost per patient	2,426 (1,455-3,734)	3,289 (2,337-4,752)	2,288 (1,366-3,503)	<0.01
Total healthcare cost				
Total cost between 2008-2015	37,685 (18,144-78,811)	70,034 (37,222-110,814)	34,325 (16,093-69,957)	<0.01
Mean total cost	60,855 ± 70,147	90,394 ±76,832	56,220 ± 67,925	<0.01
Mean annual cost per patient	8,635 ±5,591	10,227 ± 5,024	8,336 ±5,642	<0.01
Median annual cost per patient	7,506 (5,273-10,654)	9,612 (6,931-12,086)	7,149 (4,958-10,201)	<0.01

Abbreviations: medical benefits schedule (MBS), emergency department (ED), pulmonary arterial hypertension (PAH)

PAH patients who died in close temporal proximity with their hospital admission were admitted to hospital more frequently than those without PAH (3.4 (2.3–5.9) vs 2.3 (1.3–3.4), *p* = 0.001), had a higher LOS (4.3 (2.5–6.3) vs 2.5 (1.2–4.3), *p* = 0.001) and higher admission cost (AUD$5847.4 ± 3341.5 vs 4976.9 ± 2857.2, *p* = 0.05) than those who survived.

#### ED presentation and cost

During 2008–2015, 65.1% of SSc patients presented to the ED (79.7% with PAH vs 62.8% without PAH, *p* < 0.001) with an annual median per patient ED presentation of 2 (1–3) visits (Table 3). SSc-PAH patients had more frequent annual ED presentations than those without PAH (2 (1–4) vs 2 (1–3), *p* < 0.001). The most common reason for ED presentation for those with SSc-PAH was chest pain followed by dyspnea, while for those without PAH, chest pain followed by abdominal pain were the primary reasons for presentation (Table 3).

The total ED cost per patient for our cohort during 2008–2015 was AUD$3047 (1266 -5465), with a median (25th–75th) annual per patient ED presentation cost of AUD$422 (0–748) (Table 4). Again, these costs were higher for SSc-PAH patients compared with those without PAH with a total ED cost per patient of AUD$3676 (2099-5699) vs AUD$2836 (1034 – 5279), *p* < 0.001 and median (25th -75th) an annual ED cost per patient of AUD$655 (421–1009) vs AUD$411 (0–718), *p* < 0.001 (Table 4).

#### MBS utilization and cost

The majority of patients in our cohort (95.8%) utilized a MBS service during 2008–2015 (98.7% with PAH vs 95.4% without PAH, *p* = 0.05), with an annual median per patient service utilization of 76 (47–125) MBS services (Table 3). SSc-PAH patients utilized more MBS services annually than those without PAH (108 (71–162) vs 69 (43–113), *p* < 0.001). The most commonly utilized MBS services in those with and without PAH were identical with the primary services being pathology followed by medical professional visits (Table 3). Furthermore, the allied health services utilized by those with and without SSc-PAH were identical with podiatry being the most frequently utilized service followed by nurse wound care, physiotherapy and psychology sessions.

The total MBS cost per patient for our cohort during 2008–2015 was AUD$23,568 (15,987-35,111), with a median (25th -75th) annual per patient MBS cost of AUD$2426 (1455-3734) (Table 4). Again, these costs were higher for SSc-PAH patients compared with those without PAH with a total MBS cost per patient of AUD$27,531(19,493-39,738) vs AUD$22,861(15,359-33,842), *p* < 0.001 and median (25th -75th) annual MBS cost per patient of AUD$3289 (2337-4752) vs AUD$2288 (1366-3503), *p* < 0.001 (Table 4).

#### Total healthcare utilization and associated cost

The total healthcare cost (including hospital, ED and MBS cost but excluding medication cost) for our cohort during 2008–2015 was AUD$37,685 (18,144-78,811) with an annual per patient healthcare cost of AUD$7506 (5273-10,654). Total healthcare cost was higher for SSc-PAH patients compared with those without PAH with a total cost per patient of AUD$70,034 (37,222-110,814) vs AUD$34,325 (16,093 – 69,957), *p* < 0.001, and median (25th -75th) annual healthcare cost per patient of AUD$9612 (6931-12,086) vs AUD$7149 (4958-10,201), *p* < 0.001 (Table 4). In our subgroup analysis, PAH patients incurred an annual healthcare cost of AUD$1199.42 (95%CI 355.3–2043.5, *p* = 0.005) more than those without PAH matched for age at SSc onset, gender, ethnicity, disease subtype and the presence of DU, telangiectasia and calcinosis.

#### Total healthcare utilization and cost in SSc-PAH

Within three months of PAH diagnosis, over half of the SSc-PAH patients (53.3%) had utilized a healthcare service (26.7% had been admitted to hospital, 13.3% had presented to the ED and 53.3% had utilized a MBS service). The median cost per patient associated with these healthcare services was AUD$1123 (0–5383); the majority of the cost was related to hospitalizations followed by MBS service utilization. Within six months of PAH diagnosis, two-thirds of the SSc-PAH patients (66.3%) had utilized a healthcare service (30.5% had been hospitalized, 39.1% had presented to the ED department and 60.9% had utilized a MBS service). The median cost per patient for these services was AUD$2337 (0–9268). Hospitalizations were again the predominant driver of these costs followed by MBS service utilization (Table 5). By 12 months after PAH diagnosis, 89.4% of SSc patients had utilized a healthcare service (45.6% hospitalized, 40.9% presented to ED and 87.9% had utilized a MBS service) with a median cost per PAH patient of AUD$4125 (0–15,666). By 24 months post PAH diagnosis, all patients had utilized a healthcare service with a median cost per patient of AUD$11,856 (2529-28,968) with hospital admissions being the predominant driver of costs. Healthcare costs for 3, 4 and 5 years post PAH diagnosis followed a similar pattern and are summarized in Table 5. The total per patient medication cost associated with the use of PAH specific vasodilator therapy between 2008 and 2015 was AUD$53,842 (18,728-100,882) and is summarized in Additional file 1: Table S1.

**Table 5:** Per patient annum average healthcare cost (AUD$) from PAH diagnosis

Per annum cost from PAH diagnosis	Hospitalization mean±SD median (IQR)	ED Presentation mean±SD median (IQR)	Ambulatory care mean±SD median (IQR)	Total healthcare cost mean±SD median (IQR)
1^st^ year of diagnosis				
mean cost	11,016 ± 22,971	283 ± 626	3,019 ± 4,626	14,319 ± 27,040
median cost	1,008 (0-11,133)	0 (0-374)	2,458 (0-3,979)	4,125 (0-15,666)
2^nd^ year of diagnosis				
mean cost	7,240 ± 18,511	222 ± 453	2,190 ± 2,337	9,653 ± 19,833
median cost	0 (0 – 4,766)	0 (0-360)	1,740 (416-3,168)	2,737 (668 – 8,924)
3^rd^ year of diagnosis				
mean cost	6,002 ± 12,527	291 ± 640	2,029 ± 2,068	8,324 ± 13,748
median cost	0 (0-6,204)	0 (0-384)	1,554 (210 – 3,144)	3,579 (429-9,486)
4^th^ year of diagnosis				
mean cost	6,233 ± 13,729	199 ± 379	1,904 ± 2,156	8,336 ± 14,871
median cost	0 (0-6,962)	0 (0-384)	1,373 (0-2,988)	2,446 (0-10,980)
5^th^ year of diagnosis				
mean cost	5,080 ± 11,575	178 ± 433	1,890 ± 2,402	7,149 ± 12,903
median cost	0 (0-1,681)	0 (0-0)	1,178 (0-2,908)	1,755 (0-6,602)

Abbreviations: pulmonary arterial hypertension (PAH), emergency department (ED) Medical Benefit Schedule (MBS), standard deviation (SD), interquartile range (IQR)

Determinants of above median total annual healthcare cost (and its components excluding medication cost) associated with SSc-PAH by univariate logistic regression are summarized in Additional file 1: Table S2. By multivariable logistic regression, severity of PAH (encompassing WHO-FC) was the only determinant of total annual healthcare cost in SSc-PAH patients (OR 2.5, *p* = 0.03) (Table 6). After excluding medication costs, combination PAH specific therapy did not significantly impact healthcare cost compared with monotherapy alone. Determinants of each component of this healthcare cost in SSc-PAH were also assessed. In multivariable logistic regression, female gender (OR 3.1, *p* = 0.05) and the presence of severe PAH (OR 2.3, *p* = 0.04) were associated with an above median annual hospital admission cost, while the presence of COAD (OR 5.4, *p* = 0.03) was associated with an increased ED presentation cost; no specific factors were associated with increased annual MBS cost (Table 6). Furthermore, worse WHO-FC was associated with increasing total healthcare cost predominantly driven by hospitalization cost (Additional file 1 Table S3).

**Table 6:** Determinants of above median annual total healthcare cost and its components in SSc-PAH in multivariable logistic regression

Determinants of annual total healthcare cost	OR (95%CI)	p-value
Female	2.42 (0.8-7.6)	0.13
Age at onset of PAH, years	1.00 (0.9-1.0)	0.76
Caucasian ethnicity	2.89 (0.4-19.3)	0.27
Diffuse disease subtype	1.74 (0.7-4.5)	0.26
Digital ulceration	1.82 (0.8-3.9)	0.13
Mild ILD^ (FVC>70%)	1.63 (0.8-3.6)	0.23
Combination PAH specific therapy	1.01 (0.5-2.2)	0.97
Severe PAH*	2.46 (1.1-5.6)	0.03
Determinants of hospital cost	OR (95%CI)	*p*-value
Female	3.08 (0.9-9.7)	0.05
Caucasian ethnicity	1.94 (0.4-10.2)	0.43
Diffuse disease subtype	1.38 (0.6-3.4)	0.49
Severe PAH*	2.25 (1.1-5.0)	0.04
Mild ILD^ (FVC>70%)	1.97 (0.9-4.3)	0.09
Combination PAH specific therapy	1.35 (0.6-2.8)	0.43
Determinants of ED cost	OR (95%CI)	*p*-value
Female	1.38 (0.4-5.1)	0.62
Age at onset of PAH, years	1.00 (0.9-1.1)	0.72
Caucasian ethnicity	1.52 (0.2-10.6)	0.67
Diffuse disease subtype	1.26 (0.5-3.3)	0.64
Severe PAH*	1.61 (0.6-4.0)	0.31
COAD	5.44 (1.2-25.6)	0.03
Combination PAH specific therapy	0.65 (0.3-1.5)	0.31
Determinants of MBS cost	OR (95%CI)	*p*-value
Female	1.67 (0.6-4.8)	0.34
Age at onset of PAH, years	1.01 (0.9-1.1)	0.47
Caucasian ethnicity	6.89 (0.8-60.3)	0.08
Diffuse disease subtype	1.49 (0.6-3.5)	0.35
Severe PAH*	1.02 (0.5-2.1)	0.96
Combination PAH specific therapy	1.57 (0.8-3.2)	0.22

Abbreviations: pulmonary arterial hypertension (PAH), interstitial lung disease (ILD), chronic obstructive airway disease (COAD), forced vital capacity (FVC), emergency department (ED) Medical Benefit Schedule (MBS),

^ to ensure that we were only evaluating WHO Group 1 PAH in our SSc cohort, patients were excluded if they had Group 1 PAH and co-existing ILD with a FVC <70% and an abnormal high-resolution computer tomography (HRCT) of the chest. V/Q scanning was preformed to exclude pulmonary hypertension due to chronic thromboembolism

*severe PAH defined by the presence of WHO Functional Class IV, presence of a pericardial effusion, 6MWD <300m, right atrial pressure on right heart catheter of >15 and cardiac index of <2.

## Discussion

This large well-characterized linkage study, including 153 SSc-PAH patients, investigates the healthcare burden associated with SSc-PAH in SSc patients prospectively enrolled in an Australian SSc cohort database linked with hospital, ED and ambulatory care service databases. In our cohort of 1128 SSc patients, the prevalence of SSc-PAH was 13.6% over a mean follow-up of 4.6 ± 2.6 years, which is consistent with the literature [22]. SSc-PAH patients utilized significantly more healthcare resources, including hospitalization, ED presentation and ambulatory care services, than those without PAH. PAH patients were admitted to hospital annually on average 1.5 times more than those without PAH (*p* < 0.001), with the primary reason for admission being their PAH; PAH patients had a longer LOS than those without PAH (3.5 vs 1.9 days, *p* < 0.001). Similarly, SSc-PAH patients presented to the ED more frequently and utilized more ambulatory care services on an annual basis than those without PAH (3.1 vs 2.5, *p* < 0.001 and 108 vs 69, *p* < 0.001 respectively). Use of healthcare services overall, excluding medication cost, was associated with an annual excess cost per SSc-PAH patient of AUD$1891 ± 618, *p* < 0.001, predominantly driven by hospital admission costs, with an annual excess cost per SSc-PAH patient of AUD$796 ± 1378, *p* < 0.001. The cost associated with SSc-PAH occurs early post PAH diagnosis, with 66.3% of patients utilizing a healthcare service within the first six months following diagnosis, with an associated cost per patient of AUD$2337 (0–9268); 89.4% utilizing a healthcare service within the first 12 months post PAH diagnosis with an associated cost per patient of AUD$4125 (0–15,666). Severity of PAH was the main determinant of increased healthcare cost (OR2.5, *p* = 0.03) in our PAH cohort. Combination PAH specific therapy did not significantly impact healthcare cost compared with monotherapy alone.

In comparison with a US based SSc-PAH cohort (*n* = 108), who captured their PAH cohort retrospectively in a healthcare claims database between 2003 and 2014 by the International Classification of Disease-9-Clinical Modification diagnosis codes on medical claims [4], our SSc-PAH patients utilized more healthcare services with a higher percentage of patients being hospitalized by five years post PAH diagnosis (100% vs 64%). However, the total hospitalization cost was significantly lower for our SSc-PAH cohort (AUD$35,573 ± 41,154 (USD$24,537 ± 28,386) vs USD$40,957 ± 90,855). In terms of ED presentations, 78.3% of our SSc-PAH patients had presented to the ED by five years post diagnosis, which is similar to 72% reported in this US SSc-PAH cohort [4]. Again, our total ED presentation cost over this five-year period following PAH diagnosis was lower than this US cohort (AUD$1175 ± 1456 (USD$810 ± 1004) vs USD$2987 ± 10,736). In terms of ambulatory care services, 100% of SSc-PAH patients in both cohorts had utilized an ambulatory care service at five years post PAH diagnosis with comparable total ambulatory care costs of AUD$11,034 ± 8719 (USD$7610 ± 6014) vs USD$8232 ± 5260). Similarly, the mean overall healthcare cost five years post PAH diagnosis was lower for our cohort at AUD$47,783 ± 46,081 (USD$32,959 ± 31, 785) vs (USD$63,320 ± 98,531). This discrepancy in total healthcare cost is likely related to the exclusion of medication cost in our cohort, which if included would add approximately an additional annual cost per patient of AUD$35,076 (USD$24,198) for monotherapy and AUD$35,938 (USD$24,788) for combination PAH therapy, making the total healthcare cost figures more comparable. Additionally, our healthcare cost figures exclude the cost of outpatient public hospital clinic appointments as the cost of these clinics are not standardized across hospitals and could not be included in the linkage.

Similar to our cohort, over half (57%) of those with WHO Group 1 PAH included in the REVEAL registry of US patients recruited between 2006 and 2009 [23], were admitted to hospital with the main predictor of hospitalizations being PAH severity. Congestive cardiac failure (CCF) followed by the placement or removal of a central venous catheter were the most common reasons for hospitalization in the REVEAL registry, comparable to the reasons for hospitalization in our SSc-PAH cohort (PAH followed by CCF). Hospitalization was a predictor of future hospitalization and mortality, with only 25.4% of the REVEAL cohort remaining hospital free over a 3-year follow-up period [23]. The association of hospitalization and increased risk of death has been reported previously in PAH patients [24] and has been recently included as a variable in the updated REVEAL risk score calculator used to predict clinical worsening and survival in PAH patients [25].

The number of hospital admissions in our SSc-PAH cohort was higher than that of a US based general PAH patient cohort [12], with a mean of 0.4 hospitalizations and a mean LOS of 0.3 days per patient per month (pppm) in our cohort compared with 0.1 hospitalizations/ pppm and a mean average LOS of 1.0 days/pppm which may reflect the underling severe nature and poor prognosis of SSc-PAH relative to other causes of PAH [11] in addition to the advanced age with increasing numbers of co-morbidities. Interestingly, ED presentations among our SSc-PAH cohort were lower than reported in the general PAH cohort with 0.2 ED visits/pppm compared with 0.4 visits/pppm in the general PAH cohort. For PAH cohorts, the average total healthcare cost is in the range of USD$2023–$9295 pppm depending on how PAH was captured (US claims database, transthoracic echocardiogram or RHC) and whether or not PAH specific medication was included in the study [14, 26–29]. Our cohort costs (AUD$852/pppm (USD$587) are not comparable to these studies as we have a very stringently defined SSc-PAH cohort, all diagnosed on RHC, and our total healthcare costs are deliberately exclusive of PAH specific therapies to allow the cost disparity in terms of healthcare utilization between those with and without PAH to be equitable.

Strengths of our study include its well-characterized SSc-PAH population, all fulfilling the ACR/EULAR criteria for SSc [17], followed over a substantial time frame and PAH being diagnosed on RHC according to international criteria [18]. Our study is also strengthened by its linkage methodology which allows the real world reliable calculation of healthcare utilization and associated cost to the Australian government. Study limitations include the possibility of underestimating the cost associated with PAH managed primarily in the public hospital system as our data linkage methodology did not include the cost of public outpatient clinics nor did we did capture the cost of any.

privately funded allied health service not covered by the MBS. Furthermore, medication compliance was not verified in this study potentially causing a slight over estimate of medication costs if medications recorded in the database were not dispensed.

## Conclusions

Despite SSc-PAH being a low prevalence disease, it is associated with significant healthcare resource utilization and associated economic burden predominantly driven by the severity of PAH. Our study highlights the need to identify more effective therapeutic strategies to improve disease progression and management of this costly disease.

## Supplementary information

**Additional file 1: Table S1.** PAH specific vasodilator therapy cost **Table S2**. Determinants of above median annual total healthcare cost in SSc-PAH in univariant logistic regression **Table S3**. Impact of PAH severity on total healthcare cost and its components in SSc-PAH

## Data Availability

The datasets generated and/or analysed during the current study are not publicly available but are available from the corresponding author on reasonable request.

## Abbreviations

(6MWD): Six minute walk distance
(CI): Cardiac index
(COAD): Chronic obstructive airways disease
(CVA): Cerebrovascular accident
(DLCO): Diffusing capacity of the lungs for carbon monoxide
(GIT): Gastrointestinal tract
(HAQ): Health assessment questionnaire
(HTN): Hypertension
(ILD): Interstitial lung disease
(mPAP): Mean pulmonary arterial pressure
(mRAP): Mean right atrial pressure
(PAH): Pulmonary arterial hypertension
(PAWP): Pulmonary arterial wedge pressure
(PVD): Peripheral vascular disease
(PVR): Pulmonary vascular resistance
(sHAQ): Scleroderma HAQ
(SSc): Systemic sclerosis
(WHO): World health organization
DLCO: Divided by the alveolar volume.
(DLCO/VA): Medical benefits schedule (MBS)
(LOS): Length of stay
(ED): Emergency department
(LRTI): Lower respiratory tract infection
(SD): Standard deviation
(IQR): Interquartile range
