# Supplementary material for: The economic burden of systemic sclerosis related pulmonary arterial hypertension in Australia
Source: BMC Pulm Med. 2019 Nov 27;19:226. doi: 10.1186/s12890-019-0989-1 (PMC6881974; doi:10.1186/s12890-019-0989-1)
Supplement: Supplementary file 1 — Additional file 1: Table S1. PAH specific vasodilator therapy cost Table S2. Determinants of above median annual total healthcare cost in SSc-PAH in univariant logistic regression Table S3. Impact of PAH severity on total healthcare cost and its components in SSc-PAH [file 12890_2019_989_MOESM1_ESM.docx]

Supplementary Table 1. PAH specific vasodilator therapy cost

| **Total PAH specific vasodilator therapy cost** | **Average total per patient medication cost (2008-2015)** |
| --- | --- |
| Per patient medication cost (2008-2015)  Median (25-75^th^)  Mean (±SD) | 53,842 (18,728-100,882)  63,960 ± 48,174 |
| Composed of  Phosphodiesterase-5-inhibitors  Sildenafil per patient cost (n=79)  Median (25-75^th^)  Mean (±SD)  Tadalafil per patient cost (n=15)  Median (25-75^th^)  Mean (±SD)  Endothethial receptor antagonists  Bosentan per patient cost (n=117)  Median (25-75^th^)  Mean (±SD)  Macitentan per patient cost (n=12)  Median (25-75^th^)  Mean (±SD)  Ambrisentan per patient cost (n=30)  Median (25-75^th^)  Mean (±SD) | 1,784 (892-3,222)  2,302 ± 1,468  10,277 (9,153-18,123)  13,095 ± 4,727  66,535 (37,205–109,294)  73,890 ± 45,133  34,292 (32,083–38,999)  36,702 ± 7,041  79,230 (42,824-108,094)  79,753 ± 35,452 |

Abbreviations: standard deviation (SD)

Although prostacyclin analogues are available for use in severe PAH in Australia, there were no patients prescribed a prostacyclin analogue during this time period in our cohort

Supplementary Table 2. Determinants of above median annual total healthcare cost in SSc-PAH in univariant logistic regression

| **Type of cost** | **Total healthcare cost** | | **Hospital cost** | | **ED cost** | | **MBS cost** | |
| --- | --- | --- | --- | --- | --- | --- | --- | --- |
| **Patient characteristic** | **OR (95%CI)** | **p-value** | **OR (95%CI)** | **p-value** | **OR (95%CI)** | **p-value** | **OR (95%CI)** | **p-value** |
| Patient number | 153 |  | 153 |  | 153 |  | 153 |  |
| Demographics  Female  Age at onset of SSc*, years^#^  Age at onset of PAH, years^#^  Caucasian ethnicity  Diffuse disease | 1.89 (0.7-5.1)  0.99 (0.9-1.0)  0.99 (0.9-1.0)  2.69 (0.5-14.4)  1.42 (0.7-3.0) | 0.21  0.88  0.68  0.25  0.37 | 1.83 (0.7-4.9)  1.01 (0.9-1.0)  1.00 (0.9-1.0)  1.31 (0.3-6.1)  1.27 (0.6-2.7) | 0.24  0.66  0.98  0.73  0.53 | 1.33 (0.4-4.1)  0.99 (0.9-1.0)  1.00 (0.9-1.1)  0.91 (0.2-5.3)  1.24 (0.5-2.9) | 0.62 0.87  0.66  0.93  0.62 | 1.51 (0.6-3.9)  1.01 (0.9-1.0)  1.00 (0.9-1.1)  8.19 (0.9-68.3)  0.95 (0.5-1.9) | 0.41  0.35  0.59  0.05  0.89 |
| Clinical manifestations**  Digital Ulcers  GI involvement  Renal Crisis  Pericardial effusion  Mild ILD (FVC>70%) | 1.52 (0.8-2.9)  0.71 (0.3-1.9)  0.99 (0.1-16.1)  2.69 (1.1-6.7)  1.84 (0.9-3.6) | 0.23  0.48  0.99  0.03  0.08 | 1.01 (0.5-1.9)  0.53 (0.2-1.4)  -  2.78 (1.1-6.9)  1.94 (0.9-3.8) | 0.98  0.21  -  0.03  0.05 | 0.82 (0.4-1.7)  1.59 (0.5-4.7)  0.58 (0.1-9.5)  0.99 (0.4-2.5)  1.22 (0.6-2.5) | 0.61  0.41  0.70  0.97  0.60 | 0.79 (0.4-1.5)  1.69 (0.6-4.4)  -  0.96 (0.4-2.2)  1.09 (0.6-2.1) | 0.49  0.29  -  0.94  0.79 |
| Severe PAH*** | 2.16 (1.0-4.50 | 0.04 | 2.39 (1.1-5.2) | 0.02 | 1.77 (0.8-4.0) | 0.17 | 1.19 (0.6-2.4) | 0.61 |
| Co-morbidities  Angina  CVA  Diabetes Mellitus  COAD  Current smoker | 1.77 (0.8-3.9)  0.38 (0.1-2.0)  1.89 (0.6-5.9)  2.01 (0.8-5.4)  0.59 (0.2-1.9) | 0.17  0.25  0.28  0.16  0.37 | 0.93 (0.4-2.1)  0.38 (0.1-2.1)  1.39 (0.5-4.3)  0.81 (0.3-2.1)  0.61 (0.2-1.9) | 0.87  0.27  0.56  0.65  0.40 | 1.91 (0.7-4.9)  1.17 (0.2-6.7)  0.65 (0.2-2.1)  6.09 (1.3-27.7)  1.03 (0.3-3.7) | 0.18  0.85  0.47  0.02  0.97 | 1.69 (0.8-3.8)  0.71 (0.2-3.2)  1.83 (0.6-5.7)  0.71 (0.3-1.7)  0.35 (0.1-1.2) | 0.20  0.66  0.30  0.44  0.09 |
| Combination PAH therapy | 1.10 (0.6-2.2) | 0.78 | 1.66 (0.84-3.3) | 0.15 | 0.69 (0.33-1.5) | 0.33 | 1.48 (0.8-2.9) | 0.25 |

Abbreviations: pulmonary arterial hypertension (PAH), interstitial lung disease (ILD), gastrointestinal tract (GIT), six minute walk distance (6MWD), world health organization functional class (WHO FC), cerebrovascular accident (CVA), peripheral vascular disease (PVD), chronic obstructive airways disease (COAD),

* age at onset of SSc defined as from first non-Raynaud’s disease manifestation,

**clinical manifestations defined as present if ever present from SSc diagnosis

*** severe PAH defined by the presence of WHO Functional Class IV, presence of a pericardial effusion, 6MWD <300m, right atrial pressure on right heart catheter of >15 and cardiac index of <2.

Supplementary Table 3. Impact of PAH severity on total healthcare cost and its components in SSc-PAH

| **Characteristics** | **Average healthcare cost per patient per year** | **p-value** | **Hospital** | **p-value** | **ED** | **p- value** | **MBS** | **p-value** |
| --- | --- | --- | --- | --- | --- | --- | --- | --- |
| **Severe PAH** |  |  |  |  |  |  |  |  |
| Yes  Mean  Median | 10,777 ± 5,254  9,934 (7,627-12,797) | 0.02 | 5,756 ± 3,147  5,085 (3,485-7,201) | 0.01 | 920 ± 675  758 (507-1,054) | 0.01 | 4,064 ± 3.088  3,515 (2,345-4,719) | 0.82 |
| No  Mean  Median | 8,982 ± 4,252  8,472 (5,967 – 11,346) |  | 4,606 ± 3,023  3,769 (2,726 – 5,720) |  | 526 ± 579  446 (0-865) |  | 4,039 ± 3,367  2,958 (2,307 – 4,822) |  |
|  | | | | | | | | |
| **WHO FC** |  |  |  |  |  |  |  |  |
| Class I  Mean  Median | 7,173 ± 3,614  7173 (4617-9728) | 0.01 | 3,263 ± 1,944  3,263 (1,888-4,638) | 0.01 | 872 ± 45  873 (841-905) | 0.22 | 3,036 ± 1624  3,036 (1,888-4,184) | 0.21 |
| Class II  Mean  Median | 7,068 ± 2,773  6,931 (5,409 -8,206) |  | 3,418 ± 1,928  2,884 (2,007-4,484) |  | 551 ± 506  449 (0-965) |  | 4113 ± 4604  2,660 (2,162-4,046) |  |
| Class III  Mean  Median | 10,885 ± 5,799  9,975 (7,270-12,564) |  | 5,748 ± 3,371  5,052 (3,421-7,334) |  | 713 ± 694  605 (0-913) |  | 4264 ± 3347  3,644 (2,345-5,084) |  |
| Class IV  Mean  Median | 9,998 ± 3,342  9,868 (7,627-11,585) |  | 5,308 ± 2,426  4,946 (3,658-6,368) |  | 991 ± 661  790 (569-1,410) |  | 3,700 ± 2,169  3,176 (2,408-4,429) |  |
